# Dose‒response relationship between physical activity and all-cause mortality in Chinese adults

**DOI:** 10.1038/s41598-025-26356-8

**Published:** 2025-12-08

**Authors:** Feifei Wang, Yi Wang, Kaiyue Wang, Shouling Wu, Yue Chen, Yaqi Li, Shuohua Chen, Xiang Gao

**Affiliations:** 1 Faculty of Nursing, Medical college, Jiaxing University, Jiaxing, 314001 People’s Republic of China; 2 Department of Nutrition and Food Hygiene, School of Public Health, Institute of Nutrition, Zhongshan Hospital, Fudan University, Shanghai, 200032 People’s Republic of China; 3 Cardiology Department, Kailuan Majiagou Hospital, Tangshan, 063022 China; 4 Department of Cardiology, Kailuan Hospital, North China University of Science and Technology, Tangshan, 063001 China

**Keywords:** Physical activity, Mortality, Vigorous physical activity, Moderate physical activity, All-cause mortality, Dose‒response relationship, Lifestyle, Life expectancy, Diseases, Health care, Risk factors

## Abstract

To determine the dose-response association between physical activity (PA) and all-cause mortality to identify minimum PA thresholds for significant mortality risk reduction by using a nationally representative sample of Chinese adults. This population-based cohort study included 109,407 Chinese participants from the Kailuan study with linkage to national death records up to December 31, 2022. Baseline data were derived from self-reported surveys among participants aged 53.32 ± 13.55 years. During a median follow-up of 6.99 (IQR 5.99–6.99) years, 4571 participants died from all causes. Performing at least 75 min per week of physical activity contributed to significantly reduced all-cause mortality. Compared with physical inactivity, total physical activity was inversely associated with the risk of mortality in the very low (HR: 1.08; 95% CI: 0.98–1.19), low (HR: 0.89; 95% CI: 0.81–0.98), medium (HR: 0.88; 95% CI: 0.79–0.96) and high (HR: 0.77; 95% CI: 0.69–0.85) quartiles. The dose‒response curve revealed a steady decline in HRs with more minutes of physical activity per week with all-cause mortality. Our study demonstrated an inverse nonlinear dose‒response relationship between physical activity and all-cause mortality in elderly adults, with as little as 75 min of weekly activity showing clinically meaningful risk reduction compared with inactivity. These findings suggest that mortality benefits at lower thresholds for elderly adults. The results provide evidence-based justification for health policies that promote graduated physical activity targets to improve population-level adherence among aging communities.

**Supplementary Information:**

The online version contains supplementary material available at 10.1038/s41598-025-26356-8.

## Introduction

Physical activity (PA) has been recommended by the World Health Organization (WHO) for its significant health benefits for hearts, bodies, and minds in their physical activity guidelines^1^. The beneficial mechanisms of physical activity have been explained by a wide range of studies on preventing and managing noncommunicable diseases (NCDs), such as heart disease, stroke, diabetes, and several cancers^2,3^. Physical activity is also effective in reducing adiposity^4^, improving sleep quality^5^, and reducing symptoms of anxiety and depression^6^. Additionally, evidence from observational studies has demonstrated significant associations of physical activity with lower risks of subsequent mortality^7^.

The WHO’s 2020 guidelines on physical activity indicated a lack of precise dose‒response data between PA and mortality^8^. Although systematic reviews and meta-analyses have provided strong evidence that both low and high amounts of PA could reduce the risk of mortality in adults^9,10^, an existing study from a Japanese cohort revealed a nonlinear dose–response relationship^11^. Regarding the health benefits of accumulated physical activity, studies exploring the dose‒response relationship between PA minutes and mortality in China is necessary. Only one study examined the PA dose-dependent relationship with pneumonia-specific mortality among Chinese adults^12^. Unlike revealing a dose‒response relationship between PA and all-cause mortality in populations with chronic diseases^13^, our analysis excluded individuals with major comorbidities to specifically assess this association in healthier elderly adults. Considering the inevitable mortality risk associated with chronic diseases, it is essential to identify the dose‒response curve of PA in relation to all-cause mortality among the general population.

Despite established links between physical activity and mortality, evidence remains scarce for the Chinese adult population, particularly regarding precise dose‒response thresholds. To address this gap, this prospective cohort study aims to (1) quantify the nonlinear association between PA volume and all-cause mortality in a comorbidity-free Chinese population and (2) identify minimum PA thresholds for significant mortality risk reduction. By excluding major chronic diseases and employing rigorous covariate adjustment, our study provides tailored recommendations for healthy aging in China’s rapidly growing elderly population.

## Methods

### Study participants

This study utilized data from the Kailuan Study, an ongoing healthcare examination that includes surveys and health examinations conducted every 2 years since 2006. The study is funded by Tangshan Kailuan Ltd. in China. Detailed information about the Kailuan study design, survey methods, clinical examinations and participant characteristics has been described elsewhere^14,15^. Potential participants were selected via a multistage design and included a total of 109,407 adult participants selected from the years 2014 and 2016. Baseline information, including lifestyle behaviors, social status, health status, and disease history, was obtained from each registered participant.

**Fig. 1:**
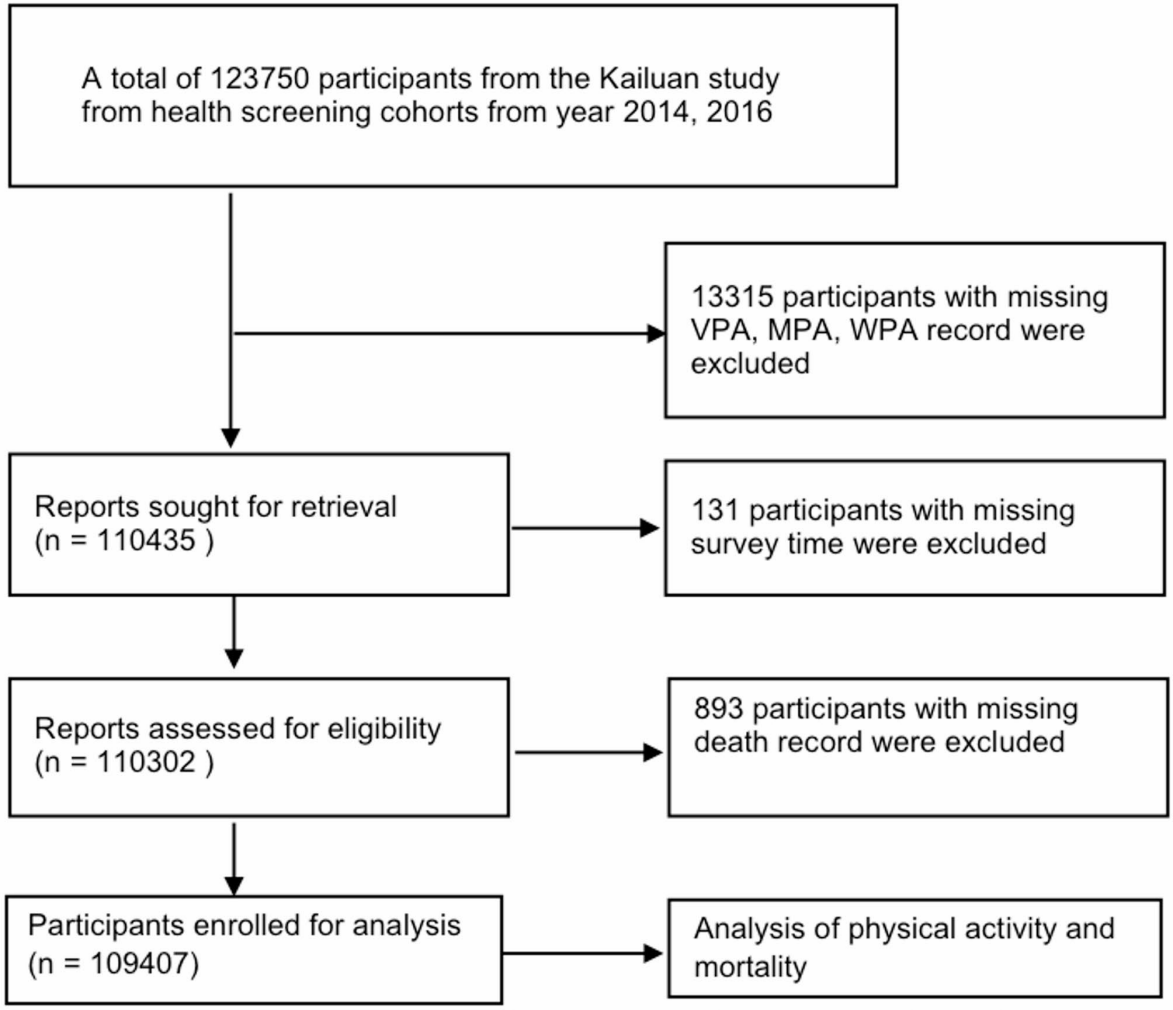
Flow diagram of study participants. Abbreviations: VPA vigorous physical activity; MPA moderate physical activity; WPA walking physical activity.

These baseline data were collected by trained doctors and nurses from 11 hospitals within the KaiLuan Group, who are responsible for healthcare. It was linked to clinically registered death records from Kailuan hospitals up to December 31, 2022. Participants with missing responses regarding physical activity habits were excluded (*n* = 13315). Additionally, participants with missing survey time data (*n* = 131) and/or missing death records (*n* = 893) were also excluded. A total of 109,407 participants were included in the final statistical analysis (Fig. 1). Similar characteristics were found between the exclude and non-exclude participants (eTable 6). The study was approved by the KaiLuan General Hospital Ethics Committee, and written informed consent was obtained from all participants. The study was performed in accordance with relevant guidelines.

### Assessment of physical activity

Physical activity was recorded by using the standardized International Physical Activity Questionnaire (IPAQ) short form. During the baseline survey, participants were asked about the frequency and duration of physical activities at different intensity levels (including vigorous physical activity, moderate physical activity and walking activity) during the past week. The frequency of physical activity was reported as days per week (“<1 day”, “1–2 days”, “3–4 days”, “5–6 days”, “every day”)^16^. The duration of physical activity was recorded in minutes of exercise (“Never”, “<30 minutes”, “30–60 minutes”, “>60 minutes”). Total physical activity (TPA) was calculated as the sum of weighted vigorous physical activity, weighted moderate physical activity and weighted walking activity by multiplying physical activity frequency and duration by weight (frequency * duration*60, frequency * duration*45, frequency * duration*15). The formula referred to an existing study using formula : “weekly total MET minutes = weekly vigorous exercise days × daily vigorous exercise duration (minutes) × (8 METs) + weekly moderate exercise days × daily moderate exercise duration (minutes) × (4 METs) + weekly walk exercise days × daily walk exercise duration (minutes) × (3.3 METs)”^17^.

### Assessment of covariates

Covariates were assessed at baseline and analyzed as time-invariant variables. Biennial demographic data, including age, sex, level of education, level of income, marital status, body height and body weight, lifestyle factors, such as smoking habits, drinking habits and use of screen devices, work-related data, such as work type and intensity, and disease history including Parkinson’s disease (PD), stroke, fatty liver, heart failure, and myocardial infarction, were collected through standardized questionnaires and clinical examinations at baseline and follow-up. For level of education, we categorized education into three levels: “illiteracy/primary school”, “middle/high school”, and “college or above”. Regarding income, we grouped participants into “<1000yuan”, “1000-3000yuan”, and “>=3000yuan”. Body height and weight were measured by trained staff using well-calibrated instruments, and body mass index (BMI) was calculated as weight in kilograms divided by height in meters squared. Smoking habits were assessed by asking about the number of cigarettes consumed per day, categorized as “<= 10 cigarettes”, “11–20 cigarettes”, or “>=20 cigarettes”. Drinking habits were divided into non-drinkers and drinkers. The participants self-reported their work type and occupational physical intensity, with work intensity classified into four categories: “no intensity”, “light intensity”, “moderate intensity”, and “heavy intensity”. Missing covariate data were handled through single imputation: continuous variables were replaced with the mean value, and categorical variables were replaced with the mode (dominant category).

### Ascertainment of mortality

All participants were followed up every two years until death or December 31, 2022. Information regarding the cause of death was obtained from Kailuan Hospital records, death certificates from provincial vital statistics offices, and medical records from medical insurance or hospitals. Death ascertains the required confirmation from ≥ 2 sources (e.g., hospital + insurance records). Discordant cases were resolved by prioritizing provincial certificates and then physician review by using standardized diagnostic criteria (i.e., ICD-10 codes).

### Statistical analysis

For baseline characteristics, continuous variables are described as medians or means with standard deviations (SEs), and categorical variables are presented as proportions (%). Comparisons of continuous or categorical variables were conducted via one-way ANOVA tests or Chi-square tests.

By categorizing participants into physically inactive and physically active groups, the physically active participants were further divided into quartiles on the basis of total physical activity volume per week (“<75 min/wk”, “75–360 min/wk”, “360–720 min/wk”, “>720 min/wk”), and characteristics were compared across these groups. Person-years were calculated from the baseline examination date to the date of death or the end of follow-up (December 31, 2022), whichever came first. We then analyzed the associations between minutes of physical activity and all-cause mortality via Cox proportional hazards models. In the initial model (Model 1), hazard ratios (HRs) and 95% confidence intervals (CIs) of mortality were estimated, adjusting for age and sex. Model 2 included additional adjustments for potential confounders such as cigarette intake, drinking habits, screen time, marital status, educational level and income level. Model 3 was further adjusted for BMI, history of Parkinson’s disease (PD), stroke, heart failure, fatty liver and myocardial infarction. Model 4 added adjustments for work intensity and work type in addition to the variables in Model 3.

To investigate the dose‒response relationship between total physical activity and the risk of all-cause mortality, we utilized a restricted cubic spline (RCS) regression with 4 knots placed at the 5th, 35th, 65th, and 95th percentiles. We employed the Akaike information criterion (AIC) to select the restricted cubic spline with 4 knots and chose the model with the lowest AIC. We designated the physically inactive group with 0 min/week of TPA as the reference group.

Subgroup analysis was performed using a forest plot. Comparisons were made between participants below and above 65 years of age, male and female, brain workers and manual workers, participants in active positions or retired, and participants with a medical history of stroke, fatty liver, or heart failure. We further examined the combined associations of age and physical activity with all-cause mortality. A heatmap was created to display the joint effects as age increased every 10 years from 18 to 99 years.

Finally, several sensitivity analyses were performed: (1) by excluding participants who died in the first two years of follow-up and removing participants with less than five years of follow-up and (2) by excluding participants with a history of stroke, heart failure or fatty liver disease. Furthermore, we examined the correlations between vigorous physical activity, moderate physical activity, and walking activity. We also investigated the potential confounding effect of BMI on physical activity for all-cause mortality. Statistical analyses were conducted using SAS (version 9.4) and R (version 4.2.3). A two-sided *P* < 0.05 was used to determine statistical significance.

## Results

### Population characteristics

In this study, 109,407 eligible adults were included, among whom 78.9% were men. At baseline, the mean age of the included participants was 53.3 (SD = 13.6) years, and the mean BMI was 24.9 (SD = 3.38) kg/m^2^. The education level and average monthly income for Chinese people were relatively low, with 85.85% having a middle or high school level of education and 73.45% earning less than 1000 Chinese yuan. The majority of participants were manual workers (89.80%), whereas only 10.20% were office workers. A total of 48.66% of people had no physical activity habits, and for those with physical activity habits, the mean duration was 224.63 min/wk. For those who were physically active, the TPA was categorized into quartiles (“0–75 min/wk”, “75–360 min/wk”, “360–720 min/wk”, “>720 min/wk”). Table 1 provides more details by quartiles of TPA durations per week.

**Table 1:** Baseline characteristics of participants by total physical activity per week (*N* = 109407).

Characteristics		Total Physical Activity per Week, Mean (SD)				Overall
	No TPA	Quartile 1 (0 < TPA < 75 min)	Quartile 2 (75 < = TPA < 360 min)	Quartile 3 (360 < = TPA < 720 min)	Quartile 4 (TPA > 720 min)	
Total participants No. (%)	53,240(48.66)	11,973(10.94)	15,043(13.75)	14,366(13.13)	14,785(13.51)	109,407(100)
Men No. (%)	40,471(76.02)	9743(81.37)	12,299(81.76)	11,626(80.93)	12,228(82.71)	86,367(78.94)
Age, year	54.54 ± 13.15	52.86 ± 14.23	51.87 ± 13.69	52.40 ± 13.51	51.69 ± 13.89	53.32 ± 13.55
BMI	24.93 ± 3.38	24.89 ± 3.37	24.90 ± 3.41	24.89 ± 3.31	24.93 ± 3.37	24.92 ± 3.38
Marriage status No. (%)						
Single	834(1.57)	438(3.66)	477(3.17)	438(3.05)	503(3.40)	2263(2.07)
Married	51,571(96.87)	11,253(93.99)	14,262(94.81)	13,568(94.45)	13,863(93.76)	104,965(95.94)
Divorced	376(0.71)	113(0.94)	121(0.80)	138(0.96)	155(1.05)	873(0.80)
Widowed	314(0.59)	119(0.99)	118(0.78)	153(1.07)	178(1.20)	920(0.84)
Re-married	145(0.27)	50(0.42)	65(0.43)	69(0.48)	86(0.58)	384(0.35)
Education level No. (%)						
Illiteracy/primary school	688(1.29)	798(6.66)	612(4.07)	397(2.76)	245(1.66)	2740(2.50)
Middle/high school	48,659(91.40)	9160(76.51)	12,002(79.78)	11,342(78.95)	12,758(86.29)	93,919(85.85)
College or above	3893(7.31)	2015(16.83)	2429(16.15)	2627(18.29)	1782(12.05)	12,746(11.65)
Income status No. (%)						
<1000 Chinese yuan	44,744(84.04)	7422(61.99)	9710(64.55)	8842(61.55)	9638(65.19)	80,354(73.45)
1000–3000 Chinese yuan	6193(11.63)	3739(31.23)	4156(27.63)	3899(27.14)	2797(18.92)	20,784(19.00)
>=3000 Chinese yuan	2303(4.33)	812(6.78)	1177(7.82)	1625(11.31)	2350(15.89)	8267(7.56)
Work type No. (%)						
Brain worker	4045(7.60)	1635(13.66)	1876(12.47)	1977(13.76)	1676(11.34)	11,156(10.20)
Manual worker	49,195(92.40)	10,338(86.34)	13,167(87.53)	12,389(86.24)	13,109(88.66)	98,249(89.80)
Cigarette intake No. (%)						
<= 10 cigarettes	52,252(98.14)	11,243(93.90)	13,960(92.80)	13,539(94.24)	13,566(91.76)	99,099(90.58)
11–20 cigarettes	819(1.54)	661(5.52)	957(6.36)	738(5.14)	998(6.75)	8974(8.20)
>= 20 cigarettes	169(0.32)	69(0.58)	126(0.84)	89(0.62)	221(1.49)	1332(1.22)
Drinking habits No. (%)						
No drinking	34,612(65.01)	8953(74.78)	11,455(76.15)	11,296(78.63)	9032(61.09)	75,347(68.87)
Drinker	18,628(34.99)	3020(25.22)	3588(23.85)	3070(21.37)	5752(38.91)	34,058(31.13)
Work intensity No. (%)						
No intensity	39,582(74.35)	6583(54.98)	9490(63.09)	8755(60.94)	9446(63.89)	73,855(67.51)
Light intensity	5696(10.70)	3019(25.22)	2730(18.15)	2817(19.61)	3176(21.48)	17,437(15.94)
Moderate intensity	5234(9.83)	1230(10.27)	1588(10.56)	1601(11.14)	1431(9.68)	11,084(10.13)
Heavy intensity	2728(5.12)	1141(9.53)	1235(8.21)	1193(8.30)	732(4.95)	7029(6.42)
Use of screen devices (hours)	2.35 ± 1.58	2.41 ± 1.54	2.33 ± 1.53	2.39 ± 1.60	2.48 ± 1.57	2.37 ± 1.57

Abbreviation: TPA Total physical activity; SD standard deviation.

Variables are displayed in percentage or mean ± SD.

### Association of total physical activity duration with mortality

During a median follow-up of 6.99 (IQR 5.99–6.99) years, 4571 participants died from all causes. Compared with those in the physically inactive group, those who engaged in physical activity for at least 75 min per week had significantly lower all-cause mortality, as indicated by adjusted hazard ratios (HRs) and 95% confidence intervals (CIs). Total physical activity showed an inverse relationship with the risk of mortality, with HRs of 1.08 (95% CI: 0.98–1.19) for the very low quartile, 0.89 (95% CI: 0.81–0.98) for the low quartile, 0.88 (95% CI: 0.79–0.96) for the medium quartile, and 0.77 (95% CI: 0.69–0.85) for the high quartile, according to adjusted Model 4 (P_trend_ <0.0001). Table 2 displays the multivariate-adjusted hazard ratios and 95% confidence intervals of TPA duration for all-cause mortality.

**Table 2:** Associations between total physical activity and all-cause mortality among 109,407 participants.

TPA groups	No. of cases(%)	No. of men/total participants	Hazard ratio (95% CI)			
			Model 1	Model 2	Model 3	Model 4
No TPA	2456(4.61)	40,471/53,240	[Reference]	[Reference]	[Reference]	[Reference]
Quartile 1 (0 < TPA < 75 min)	592(4.94)	9743/11,973	1.09 (1.01–1.20)	1.08 (0.99–1.19)	1.07 (0.98–1.18)	1.08 (0.98–1.19)
Quartile 2 (75 < = TPA < 360 min)	530(3.52)	12,299/15,043	0.89 (0.81–0.98)	0.89 (0.81–0.98)	0.88 (0.80–0.97)	0.89 (0.81–0.98)
Quartile 3 (360 < = TPA < 720 min)	524(3.65)	11,626/14,366	0.87 (0.79–0.96)	0.86 (0.78–0.95)	0.86 (0.78–0.95)	0.88 (0.79–0.96)
Quartile 4 (TPA > 720 min)	469(3.17)	12,228/14,785	0.81 (0.73–0.89)	0.77 (0.69–0.85)	0.77 (0.69–0.85)	0.77 (0.69–0.85)
P value for trend			< 0.0001	< 0.0001	< 0.0001	< 0.0001

Abbreviation: TPA total physical activity; CI confidence interval.

Model 1 adjusted for age and gender. Model 2 adjusted for Model 1 variables plus cigarette intake; drinking habits; screen time, marriage status, education level, income level. Model 3 adjusted for Model 2 variables plus body mass index (calculated as weight in kilograms divided by height in meters squared); history of Parkinson disease, heart failure, stroke, fatty liver, myocardial infarction. Model 4 adjusted Model 3 variables plus work intensity and work type.

**Fig. 2:**
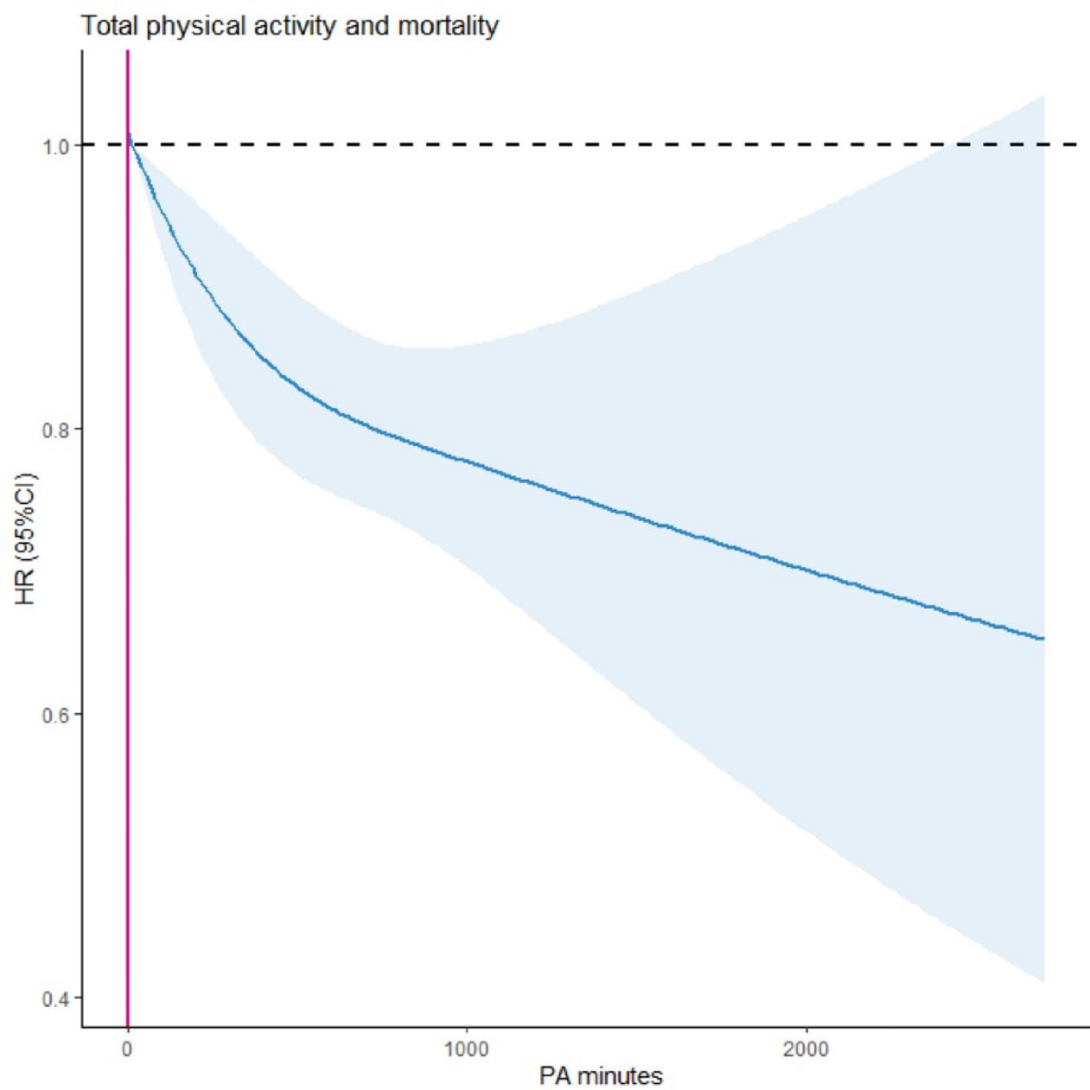
Dose-response association between total physical activity per week and all-cause mortality. The y-axis with the shared area representing hazard ratios and 95%CIs. The reference group is physically inactive participants (no PA). Model is adjusted for age, gender, cigarette intake; drinking habits; screen time, marriage status, education level, income level, body mass index (calculated as weight in kilograms divided by height in meters squared), history of Parkinson disease, heart failure, stroke, fatty liver, myocardial infarction plus work intensity and work type.

Total physical activity volume per week was associated with a lower risk of mortality when adjusted for sociodemographic factors, lifestyle factors and disease history. According to the analysis using penalized splines, participants who engaged in any amount of TPA had a lower risk of mortality than those who did not engage in any TPA. There was a steady decline in hazard ratios (HRs) with more minutes of TPA per week, up until approximately 2400 min/wk, in relation to all-cause mortality in the fully adjusted model (Fig. 2). The results indicated increased uncertainty beyond 2400 min/wk, as shown by the wide 95% confidence intervals (CIs). At higher levels of TPA, the curve showed non-significant HRs and wide CIs (Fig. 2).

### Subgroup analysis

In the subgroup analysis stratified by these covariates (see eFig. 1), the effect size of TPA on mortality was similar among participants over 65 years of age (HR: 0.91, 95% CI: 0.88–0.94) and participants under 65 years of age (HR: 0.92, 95% CI: 0.90–0.95). Non-significant differences were found for sex (men vs. women), work type (white-collar vs. blue-collar), and history of stroke, fatty liver, and heart failure (eFig. 1). However, work status (employed vs. retired) showed a significant difference in mortality (P for interaction: 0.004). We also examined the combined effect of age and the TPA on all-cause mortality, revealing that as age increased, the association between the TPA and all-cause mortality strengthened (eFig. 2).

### Sensitivity analysis

Sensitivity analyses were conducted to confirm the findings. First, after excluding participants who died in the first two years of follow-up, moderate to high levels of TPA were significantly associated with all-cause mortality (HRs: 0.88, 95% CIs: 0.79–0.98 for medium; HRs: 0.84, 95% CIs: 0.75–0.94 for high), with a P_trend_ < 0.0001 (see eTable 1 for details). Second, after excluding participants with PD, stroke, heart failure, fatty liver and myocardial infarction, the results remained consistent. TPA at low (HRs: 0.86, 95% CIs: 0.76–0.97), medium (HRs: 0.89, 95% CIs: 0.79–1.01), and high levels (HRs: 0.74, 95% CIs: 0.64–0.85) was significantly associated with all-cause mortality (P_trend_ < 0.0001) (see eTable 2 for details). When participants who were followed for less than five years were removed, the mortality rate was lower than that in the main analyses, and the associations between TPA and mortality were still consistent with those in the main analyses (eTable 5). Supportive sensitivity analysis was performed by calculating metabolic equivalent minutes (MET-minutes) to examine the dose response relationship between physical activity and mortality, the results showed that low MET-minutes was positively associated with mortality, with the increase of MET-minutes, mortality decreases significantly (eTable 7). We also examined the collinearity between vigorous physical activity, moderate physical activity, and walking activity. The results showed a strong collinearity between vigorous and moderate physical activity (β = 0.68), while walking exercise had low collinearity with both vigorous (β = 0.31) and moderate (β = 0.33) physical activity (see eTable 3 for details). Furthermore, additional analyses were conducted to investigate the associations between vigorous physical activity, moderate physical activity, walking activity, BMI, and call-cause mortality. No significant associations between BMI, TPA, and mortality were observed (see eTable 4 for details).

## Discussion

In this large prospective study, we included a sample of 109,407 Chinese adults with a mean age of 53.3 years. We found that those who engaged in a total physical activity (>75 min/wk) had a reduced risk of all-cause mortality. Our findings suggest that even a small amount of physical activity benefits mortality reduction, which aligns with global guidelines^1^. Specially for frail individuals, even a lower dose of physical activity may be beneficial^18^. The dose‒response relationship, observed L-shaped curves in studies conducted among older adults^19^. We found a steady decline in mortality rates, with more PA minutes accrued, up to approximately 2,400 min/wk, beyond which rates leveled off. After accounting for age, the associations between weekly PA minutes and all-cause mortality increased, suggesting that age is an important covariate in this population.

Many previous studies have examined the associations between PA and all-cause mortality. A cohort study involving 272,550 participants examined the amount of physical activity in metabolic equivalent of task (MET) hours per week. The study found significant associations between engaging in at least 15 MET hours (approximately 300 min/wk) of any activity and the risk of mortality in older individuals^20^. A harmonized meta-analysis including over 1 million men and women reported that high levels of moderate-intensity physical activity (approximately 60–75 min per day) appear to reduce the increased risk of death^21^. A systematic review and meta-analysis compiled evidence suggesting that higher levels of physical activity are linked to a lower risk of all-cause mortality. The pooled hazard ratios for all-cause mortality were 0.42 (CI = 0.34, 0.53) for total physical activity, 0.43 (CI = 0.35, 0.53) for moderate-to-vigorous physical activity, and 0.58 (CI = 0.43, 0.80) for light physical activity^22^.

The present study provides a unique contribution to the dose‒response relationship between total physical activity duration and all-cause mortality, as limited data are available for the Chinese population. When finer gradations were analyzed via spline analysis, the dose‒response curve was J-shaped from a study conducted in Japan^11^. However, the dose‒response curves varied slightly due to measurement error and the different baseline characteristics of the participants. Discrepancies in curve shapes may reflect methodological differences in PA assessment and/or population characteristics. For example, one study reported an L-shaped curve among participants with major chronic disease^23^. A single measure of PA at baseline was likely to show an inverted J-shaped or L-shaped dose‒response association^24^. The J-shaped patterns often emerge in younger/healthy adults^25^, whereas L-shaped curves typically dominate older populations^26^. This is because younger population almost have higher level of PA compare to older adults.

In the present study, we determined the maximum safe cutoff points for physical activity. The most commonly used cutoff value for moderate PA is 150 min/wk, and for vigorous PA, it is 75 min/wk^27,28^. Scott and colleagues published a study in The Lancet with thresholds of 150–750 min/wk (moderate PA) and >750 min/wk (high physical activity), which revealed significant associations with a graded reduction in mortality (HRs: 0.80, 95% CI 0.74–0.87 and 0.65, 0.60–0.71)^29^. Another study suggested that the most effective hypothetical volume of physical activity was to increase weekly PA to >300 min (risk ratio (RR), 0.66 (0.46–0.86))^30^. Our findings particularly identified 75 to 360 min/wk, 260 to 720 min/wk and >720 min/wk as significant thresholds for PA and all-cause mortality.

Previous studies have identified various factors related to physical activity and its impact on all-cause mortality, with age being a significant factor. Existing research has emphasized the connection between physical activity and mortality in middle-aged and older adults^7^. Compared with low levels of PA, moderate to high levels of PA have been linked to increased life expectancy, with a median age of 58 years^31^. Additionally, engaging in physical activity early in life has been shown to decrease the risk of all-cause mortality^32^. Diet is a known modifier of health outcomes and may interact with physical activity (e.g., attenuating or amplifying effects)^33^. While our results are robust across multiple sensitivity analyses, residual confounding from unmeasured variables (e.g., diet, socioeconomic status) may persist.

The present study has several strengths. This was a prospective cohort study in which the association between physical activity and the risk of mortality could be determined in the Chinese population. Potential confounding factors of physical activity and mortality were adjusted for in both the main analysis and sensitivity analysis. Data collection and process control were performed under rigid supervision.

Limitations warrant disclosure. First, the PA data collection was derived from a self-reported survey, of which potential recall bias was considered. Additionally, single-item self-report measures generally underestimate sedentary time compared with device measures^34^. Second, our study participants were predominantly older male adults, which may limit the generalizability of the findings to other age groups. Third, even though the number of included participants was relatively large, the results may not be fully representative of the entire Chinese population. The characteristics of the participants in our study represent only the low to middle income of Chinese society^15^. Fourth, information on cause-specific mortality was not available, so caution is advised when interpreting our results, as certain diseases or conditions may be closely linked to physical activity ability. Fifth, our study excluded 14,343 (11.59%) participants due to missing data, which may limit the generalizability of our findings. Furthermore, nearly half of the included population reported no activity, indicating that half of the total population was physically inactive. Previous studies have shown that the association between physical activity and all-cause mortality may vary among physically active and physically inactive individuals^35^. Although our inactivity prevalence is a bit higher than the national benchmarks, residual misclassification may exist owing to self-reported measurement of physical activity.

## Conclusions

Higher levels of total TPA reduced the risk of all-cause mortality by at least 75 min per week, following an inversely nonlinear dose‒response pattern. This study demonstrated that engaging in physical activity can greatly reduce the risk of all-cause mortality, and even low doses of physical activity significantly reduce all-cause mortality risk. These findings support public health strategies that promote incremental increases in movement, particularly for elderly populations. This study supports a paradigm shift in preventive health messaging toward inclusive, achievable physical activity targets while maintaining evidence-based recommendations for optimal duration and intensity.

## Supplementary Information

Below is the link to the electronic supplementary material.

Supplementary Material 1

## Data Availability

Data available on request from the first author FW.
