# Supplementary material for: Dose‒response relationship between physical activity and all-cause mortality in Chinese adults
Source: Sci Rep. 2025 Dec 8;15:43359. doi: 10.1038/s41598-025-26356-8 (PMC12686392; doi:10.1038/s41598-025-26356-8)
Supplement: Supplementary file 1 — Supplementary Material 1 [file 41598_2025_26356_MOESM1_ESM.docx]

**Supplemental Tables and Figures**

**Dose-response Relationship between Physical Activity and All-cause Mortality in Chinese Adults**

**eFig.1** Subgroup analysis of associations between total physical activity and call-cause mortality. *Abbreviations: HR hazard ratio; CI confidence interval; FL fat liver; HF heart failure*

**eFig.2** Heatmap of age and total physical activity per week with mortality

**eTable 1.** Associations between total physical activity and all-cause mortality excluding the first two year of follow-up

**eTable 2** Associations between total physical activity and all-cause mortality excluding participants with PD, stroke, heart failure, fat liver and myocardial infarction

**eTable 3** Correlated associations of moderate, walk and vigorous physical activity

**eTable 4** Associations of moderate, walk and vigorous physical activity with BMI for all-cause mortality

**eTable 5** Associations between total physical activity and all-cause mortality excluding the participants with less than 5 year of follow-up (n=84837)

**eTable 6** Characteristics between of exclude and non-exclude participants

**eTable 7** Associations between total MET-minutes and all-cause mortality


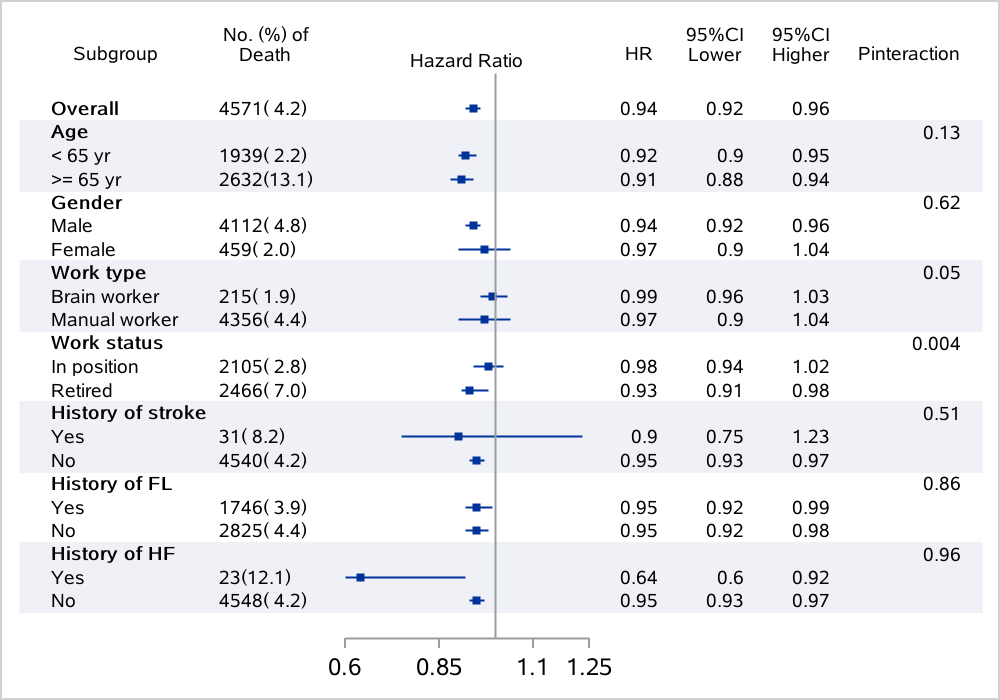


**eFig. 1** Subgroup analysis of associations between total physical activity and call-cause mortality. *Abbreviations: HR hazard ratio; CI confidence interval; FL fat liver; HF heart failure*

**eFig.2** Heatmap of age and total physical activity per week with mortality

**eTable 1**. Associations between total physical activity and all-cause mortality excluding the first two year of follow-up (n=108600)

| **TPA groups** | **No. of cases(%)** | **No. of men/total participants** | **Hazard ratio (95% CI)** | | | |
| --- | --- | --- | --- | --- | --- | --- |
|  |  |  | **Model 1** | **Model 2** | **Model 3** | **Model 4** |
| No TPA | 1977(3.75) | 12720/52761 | [Reference] | [Reference] | [Reference] | [Reference] |
| Quartile 1  (0< TPA <75min) | 495(4.17) | 2224/11876 | 1.12(1.02 – 1.24) | 1.08 (0.98 -1.21) | 1.11 (1.00 -1.23) | 1.12(1.01 – 1.24) |
| Quartile 2  (75<= TPA <360min) | 453(3.03) | 2740/14966 | 0.94 (0.85 -1.04) | 0.92 (0.83 -1.02) | 0.93 (0.84 -1.04) | 0.94 (0.84 -1.04) |
| Quartile 3  (360<= TPA <720min) | 432(3.03) | 2731/14274 | 0.89 (0.80 – 0.99) | 0.86 (0.78 -0.96) | 0.88 (0.79 -0.97) | 0.88 (0.79 – 0.98) |
| Quartile 4  (TPA >720min) | 407(2.76) | 2550/14722 | 0.87 (0.78 – 0.97) | 0.83 (0.75 -0.93) | 0.84 (0.75 -0.94) | 0.84 (0.75 -0.94) |
| P value for trend |  |  | .0009 | <.0001 | <.0001 | <.0001 |

Abbreviation: TPA Total physical activity; CI confidence interval

Model 1 adjusted for age and gender. Model 2 adjusted for Model 1 variables plus cigarette intake; drinking habits; screen time, marriage status, education level, income level. Model 3 adjusted for Model 2 variables plus body mass index (calculated as weight in kilograms divided by height in meters squared); history of heart failure, stroke, fatty liver. Model 4 adjusted Model 3 variables plus work intensity and work type.

**eTable 2** Associations between total physical activity and all-cause mortality excluding participants with PD, stroke, heart failure, fat liver and myocardial infarction (n=62943)

| **TPA groups** | **No. of cases(%)** | **No. of men/total participants** | **Hazard ratio (95% CI)** | | | |
| --- | --- | --- | --- | --- | --- | --- |
|  |  |  | **Model 1** | **Model 2** | **Model 3** | **Model 4** |
| No TPA | 1530(4.92) | 23198/31110 | [Reference] | [Reference] | [Reference] | [Reference] |
| Quartile 1  (0< TPA <75min) | 409(5.59) | 5835/7319 | 1.10 (0.99 – 1.23) | 1.07 (0.96 – 1.21) | 1.07 (0.96 – 1.21) | 1.07 (0.95 – 1.21) |
| Quartile 2  (75<= TPA <360min) | 325(3.69) | 7102/8814 | 0.86 (0.76– 0.97) | 0.85 (0.75 – 0.97) | 0.86 (075 –0.97) | 0.86 (0.76 – 0.97) |
| Quartile 3  (360<= TPA <720min) | 304(3.77) | 6407/8066 | 0.88 (0.78 – 1.00) | 0.88 (0.77 – 0.99) | 0.88 (0.78 – 0.99) | 0.89 (0.79 – 1.01) |
| Quartile 4  (TPA >720min) | 235(3.08) | 6281/7634 | 0.78 (0.68 – 0.89) | 0.74 (0.64 – 0.85) | 0.74 (0.64 – 0.86) | 0.74 (0.64 – 0.85) |
| P value for trend |  |  | <.0001 | <.0001 | <.0001 | <.0001 |

Abbreviation: TPA Total physical activity; CI confidence interval

Model 1 adjusted for age and gender. Model 2 adjusted for Model 1 variables plus cigarette intake; drinking habits; screen time, marriage status, education level, income level. Model 3 adjusted for Model 2 variables plus body mass index (calculated as weight in kilograms divided by height in meters squared). Model 4 adjusted Model 3 variables plus work intensity and work type.

**eTable 3** Correlated associations of moderate, walk and vigorous physical activity

|  | VPA | MPA | WPA |
| --- | --- | --- | --- |
| VPA | 1 | 0.68 | 0.31 |
| MPA | 0.68 | 1 | 0.35 |
| WPA | 0.31 | 0.35 | 1 |

Abbreviation: VPA vigorous physical activity; MPA moderate physical activity; WPA walking physical activity

**eTable 4** Associations of moderate, walk and vigorous physical activity with BMI for all-cause mortality

|  | Hazard ratio (95% CI) ^a^ | | |
| --- | --- | --- | --- |
|  | MPA | VPA | WPA |
| Low weight | 0.97 (0.68 – 1.39) | 1.07 (0.73 – 1.56) | 1.30 (1.09 – 1.57) |
| Normal weight | 1.06 (0.97 – 1.15) | 0.92 (0.85 – 0.99) | 1.06 (1.02 – 1.11) |
| Over weight | 1.01 (0.95 – 1.09) | 0.94 (0.88 – 1.00) | 1.11 (1.07 – 1.15) |
| Obesity | 1.24 (0.96 – 1.60) | 0.91 (0.73 – 1.14) | 1.02 (0.92 – 1.12) |

Abbreviation: CI confidence interval; MPA moderate physical activity; VPA vigorous physical activity; WPA walking physical activity; BMI body mass index

a. Model adjusted for age; gender; cigarette intake; drinking habits; screen time, marriage status, education level, income level; BMI; history of Parkinson disease, heart failure, stroke, fatty liver, myocardial infarction; work intensity and work type.

**eTable 5** Associations between total physical activity and all-cause mortality excluding the participants with less than 5 year of follow-up (n=84837)

| **TPA groups** | **No. of cases(%)** | **No. of men/total participants** | **Hazard ratio (95% CI)** | | | |
| --- | --- | --- | --- | --- | --- | --- |
|  |  |  | **Model 1** | **Model 2** | **Model 3** | **Model 4** |
| No TPA | 761(1.88) | 30551/40505 | [Reference] | [Reference] | [Reference] | [Reference] |
| Quartile 1  (0< TPA <75min) | 173(1.87) | 7499/9248 | 1.02(0.86-1.20) | 0.96(0.81-1.15) | 0.94(0.81-1.14) | 0.98(0.83-1.16) |
| Quartile 2  (75<= TPA <360min) | 177(1.48) | 9808/11982 | 0.96(0.82-1.14) | 0.93(0.78-1.09) | 0.92(0.78-1.09) | 0.93(0.79-1.11) |
| Quartile 3  (360<= TPA <720min) | 160(1.35) | 9499/11821 | 0.84(0.71-0.99) | 0.81(0.68-0.96) | 0.81(0.67-0.96) | 0.81(0.68-0.97) |
| Quartile 4  (TPA >720min) | 154(1.37) | 9351/11281 | 0.88(0.74-1.05) | 0.84(0.70-1.00) | 0.84(0.70-1.00) | 0.85(0.71-1.02) |
| P value for trend |  |  | 0.03 | 0.009 | 0.007 | 0.01 |

Abbreviation: TPA Total physical activity; CI confidence interval

Model 1 adjusted for age and gender. Model 2 adjusted for Model 1 variables plus cigarette intake; drinking habits; screen time, marriage status, education level, income level. Model 3 adjusted for Model 2 variables plus body mass index (calculated as weight in kilograms divided by height in meters squared). Model 4 adjusted Model 3 variables plus work intensity and work type.

**eTable 6** Characteristics between of exclude and non-exclude participants

|  | **Exclude participants** | **Non-exclude participants** |
| --- | --- | --- |
| **Total participants No. (%)** | 109407(100) | 123750(100) |
| **Men No. (%)** | 86367(78.94) | 98068(79.25) |
| **Age, year** | 53.32±13.55 | 52.79±25.80 |
| **BMI** | 24.92±3.38 | 24.97±3.43 |
| **Marriage status No. (%)** |  |  |
| Single | 2263(2.07) | 3138(2.54) |
| Married | 104965(95.94) | 118137(95.46) |
| Divorced | 873(0.80) | 1027(0.83) |
| Widowed | 920(0.84) | 979(0.79) |
| Re-married | 384(0.35) | 469(0.38) |
| **Education level No. (%)** |  |  |
| Illiteracy/primary school | 2740(2.50) | 3330(2.69) |
| Middle/high school | 93919(85.85) | 105492(85.25) |
| College or above | 12746(11.65) | 14828(12.06) |
| **Income status No. (%)** |  |  |
| <1000 Chinese yuan | 80354(73.45) | 90405(73.05) |
| 1000-3000 Chinese yuan | 20784(19.00) | 24099(19.47) |
| >=3000 Chinese yuan | 8267(7.56) | 9246(7.47) |
| **Work type No. (%)** |  |  |
| Brain worker | 11156(10.20) | 12061(9.75) |
| Manual worker | 98249(89.80) | 111689(90.25) |
| **Cigarette intake No. (%)** |  |  |
| <= 10 cigarettes | 99099(90.58) | 113266(91.53) |
| 11-20 cigarettes | 8974(8.20) | 9111(7.36) |
| >= 20 cigarettes | 1332(1.22) | 1373(1.11) |
| **Drinking habits No. (%)** |  |  |
| No drinking | 75347(68.87) | 85395(69.01) |
| Drinker | 34058(31.13) | 38355(30.99) |
| **Work intensity No. (%)** |  |  |
| No intensity | 73855(67.51) | 82182(66.41) |
| Light intensity | 17437(15.94) | 19397(15.67) |
| Moderate intensity | 11084(10.13) | 12985(10.49) |
| Heavy intensity | 7029(6.42) | 9186(7.42) |
| **Use of screen devices (hours)** | 2.37±1.57 | 2.38±1.60 |

**eTable 7** Associations between total MET-minutes and all-cause mortality (n=89289)

| **MET groups (minute)** | **No. of cases (%)** | **No. of men/total participants** | **Hazard ratio (95% CI)** | | | |
| --- | --- | --- | --- | --- | --- | --- |
|  |  |  | **Model 1** | **Model 2** | **Model 3** | **Model 4** |
| Quartile 1  (0< MET < 16.5) | 22.65 | 9069/10897 | [Reference] | [Reference] | [Reference] | [Reference] |
| Quartile 2  (16.5 <= MET <59.4) | 25.61 | 10401/12320 | 1.07(1.04-1.09) | 1.09(0.07-1.12) | 1.09(1.06-1.12) | 1.09(1.06-1.12) |
| Quartile 3  (59.4<= MET <119.4) | 26.71 | 10134/12851 | 1.00(0.97-1.03) | 1.04(1.02-1.07) | 1.04(1.02-1.07) | 1.04(1.01-1.06) |
| Quartile 4  (MET >119.4) | 25.03 | 10056/12040 | 0.94(0.91-0.96) | 0.87(0.85-0.89) | 0.87(0.85-0.89) | 0.86(0.84-0.88) |
| P value for trend |  |  | <.0001 | <.0001 | <.0001 | <.0001 |

MET: metabolic equivalent

Model 1 adjusted for age and gender. Model 2 adjusted for Model 1 variables plus cigarette intake; drinking habits; screen time, marriage status, education level, income level. Model 3 adjusted for Model 2 variables plus body mass index (calculated as weight in kilograms divided by height in meters squared); history of Parkinson disease, heart failure, stroke, fatty liver, myocardial infarction. Model 4 adjusted Model 3 variables plus work intensity and work type.
